# Morphometric Analysis and Classification of the Cross-Sectional Shape of the C2 Lamina

**DOI:** 10.1155/2017/7276946

**Published:** 2017-09-25

**Authors:** Soyeon Kim, Dai-Soon Kwak, In-Beom Kim

**Affiliations:** Catholic Institute for Applied Anatomy, Department of Anatomy, College of Medicine, The Catholic University of Korea, 222 Banpo-daero, Seocho-gu, Seoul 06591, Republic of Korea

## Abstract

A thorough understanding of the morphology of the lamina of the second cervical vertebra (C2) is important for safe C2 translaminar screw placement. Although anatomical characteristics of the C2 lamina have been widely documented, individual differences in morphology have not been addressed. The aim of this study was to morphometrically analyze the cross-sectional shape of the C2 lamina and classify the shape to describe individual differences. Morphometric analysis was conducted on 145 three-dimensional C2 models based on computerized tomography images from Korean adult cadavers. Several parameters were measured on a cross-section image of the lamina model. Based on numerical criteria, all of the C2 lamina's cross-sectional shapes could be categorized into three distinctive morphological types: pyriform, ellipse, and obpyriform shapes. We confirmed that most Koreans can accommodate C2 translaminar screw placement with a lower limit of the 95% confidence interval of thickness measured at 6.26 mm. Morphometric analysis suggested that the obpyriform-shaped lamina (4.48%) is likely to require screw trajectory adjustment to avoid cortical breakout of the screw. Our results will enhance current anatomical understanding of the C2 lamina and thus facilitate safer C2 translaminar screw placement.

## 1. Introduction

The axis is the second vertebra (C2) in the upper cervical spine that contributes to head and neck movement. The C2 has unique anatomical features, such as the dens (odontoid process) and superior articulating facets. The lamina of the C2 is reported to be the largest in the cervical spine and is often used during translaminar fixation to correct atlantoaxial or occipitocervical instability [1, 2]. Screws are inserted into the laminas of the C2 bilaterally to fuse the level with the relevant upper or lower vertebrae [2, 3]. Other options for cervical instrumentation include transarticular screw placement and C2 pars/pedicle screw fixation. Despite their biomechanical competitiveness, these fixation techniques entail a high risk of vertebral artery injury due to their close proximity to the screw path [4–7]. In 2004, Wright [2] proposed translaminar screw fixation as an alternative technique for posterior atlantoaxial arthrodesis because of relatively good visibility of relevant structures during the procedure.

Since the introduction of the translaminar screw technique by Wright, a number of studies have assessed the morphology of the C2 lamina across various populations [8–18]. Laminas are generally described as flat, bilateral plates. However, Wang [9] noted large morphological differences in C2 lamina among individuals. Conducting the translaminar screw fixation technique without consideration of individual structural differences can result in screw breakout into the dorsal or ventral space, which can increase the risk of postoperative cervical instability or neurological complications [19–21]. It is, therefore, expected that a study of the three-dimensional (3D) structure of the C2 lamina would enhance the current anatomical understanding, thus facilitating safer C2 translaminar screw placement. The 3D structure of the lamina can be delineated with a section image that is perpendicular to the longitudinal axis of the lamina. Therefore, we examined the morphological characteristics of the C2 lamina by analyzing the cross-sectional shape of 3D lamina models generated from computerized tomography (CT) images. Four anatomical parameters related to the section shape of the C2 lamina were investigated, and the section shapes were categorized so that the varied section shapes among individuals can be best explained in relation to the safe screw placement.

## 2. Materials and Methods

CT images of the cervical vertebrae of Korean cadavers were randomly selected from the database of the Catholic Digital Human Library. The Catholic Digital Human Library stores scanned CT images of cadavers that were donated to the Catholic University of Korea. Each image contained demographic information such as age and gender; however, personal information that can be linked to specific individuals was unavailable [22, 23]. CT images had 0.6 mm or 0.8 mm slice thickness and 0.391 mm to 0.461 mm pixel dimensions (SOMATOM Definition AS +, Siemens Healthcare, Germany). CT scans were performed with a plastic ball of known size (diameter: 2.25 inches) positioned alongside the cadavers for size calibration in order to ensure that the reconstructed 3D models represented the size of the real bone [23–25]. One hundred forty-five CT scans of the C2 were retrieved that had intact lamina with no signs of surgical instrumentation and no deformities on the bone. Sixty-five specimens were from females with an average age of 49 (21–96) years, and eighty specimens were from males with an average age of 54.5 (20–95) years.

**Figure 1:**
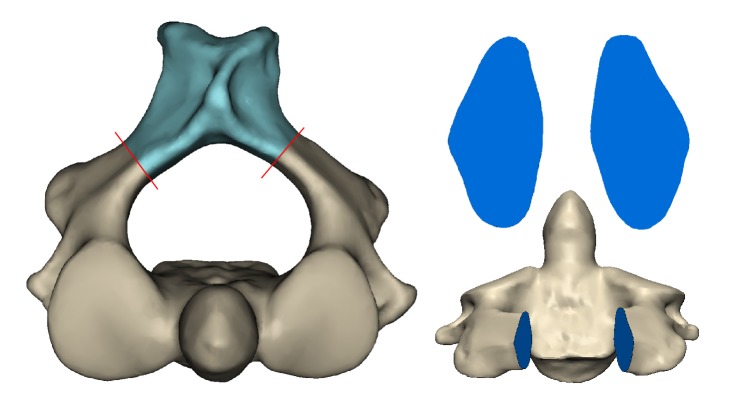
Acquisition of section shapes of the lamina on a 3D model of the C2.

**Figure 2:**
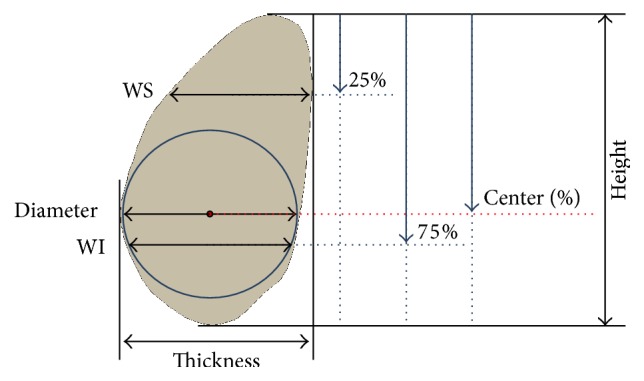
Measurement parameters, WS: width at 25% of the height from the top of the lamina; WI: width at 75% of the height from the top of the lamina.

A 3D modeling software (Mimics Ver. 19, Materialise, Belgium) enabled reconstruction of 3D models of the axis and extraction of bilateral section shapes of the lamina. The 3D models of the lamina were cut perpendicular to the longitudinal axis of the lamina at the narrowest portion on the axial view, as this portion dictates safe translaminar screw placement (Figure 1). Two observers separately adjusted the anatomical alignment of the 3D models of the C2 on Mimics software and made an orthogonal cut on the axial view. One observer repeated the alignment and cutting process to ensure intraobserver reliability. A scientific programming language (MatLab, R2016, MathWorks, MA) automatically measured variables, including thickness, height, diameter, the center position of a maximally applicable circle, and width at 25% (WS) and 75% (WI) of height from the top of the lamina, on the extracted section shape of the lamina (Figure 2). MatLab was programmed to automatically draw a maximally applicable circle that did not break out of the outline of the laminar section on the sectional image. Therefore, the diameter and the center position were expected to more precisely describe the width and position of the safest part of the lamina for the screw to pass through without penetrating cortical bone. All measurement parameters were automatically calculated with certain landmark points on the section images, and two observers assessed correct alignment and landmark points created through the program.

**Figure 3:**
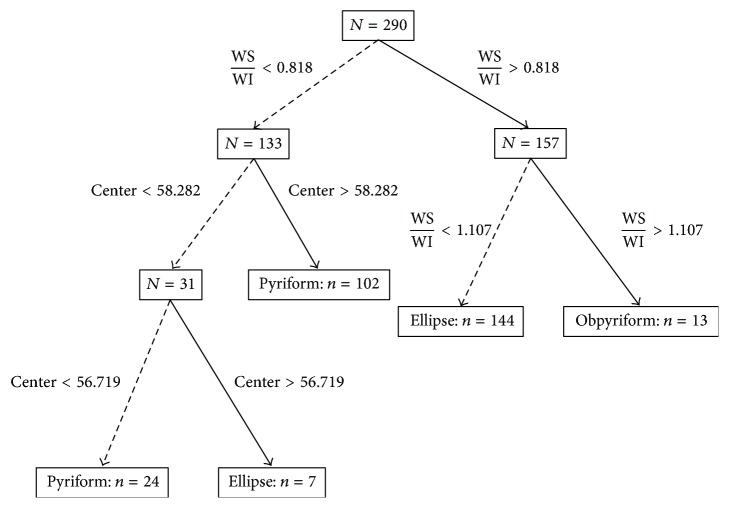
Classification tree for determination of section shape type, WS: width at 25% of the height from the top of the lamina; WI: width at 75% of the height from the top of the lamina.

The ratio of WS to WI (WS/WI) and the center position values were used to analyze the cross-sectional shape of the C2 in relation to the part of the lamina within which a screw with the maximum diameter could be safely located. A classification tree was created using the Classification Tree Package via R (Ver. 1.0.44, RStudio, Inc.) to determine cutoff values, thus excluding subjectivity (Figure 3). Average differences were compared with Student's *t*-test and ANOVA.

## 3. Results

The laminar section had thickness of 6.82 ± 1.25 mm and height of 13.46 ± 1.5 mm (Table 1). The diameter of the maximally applicable circle was 6.40 ± 1.21 mm. A significant difference was noted between the values of WS and WI (*p* < 0.001); the WS was 4.69 ± 1.15 mm and the WI was 5.73 ± 1.19 mm. On average, the center of the maximally applicable circle was positioned at 57.31% of the lamina's height from the top. Differences between the sexes were found in the three parameters of thickness, height, and diameter, with larger dimensions in males (*p* < 0.001). For the four measurement variables thickness, height, diameter, and center position, intraclass correlation coefficients (ICCs) revealed excellent interobserver reliability (ICCs = 0.943~0.989) and intraobserver reliability (ICCs = 0.936~0.990).

**Figure 4:**
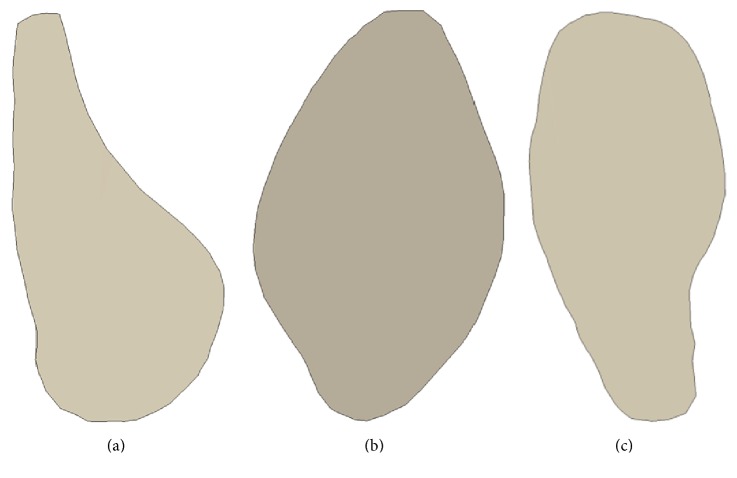
Types of cross-section shapes of the lamina: (a) pyriform shape (43.45%); (b) elliptical shape (52.07%); (c) obpyriform shape (4.48%).

The classified cross-sectional shapes in relation to the location of the screw with the maximum diameter were named as obpyriform, pyriform, and ellipse according to the characteristics of each segregated group (Figure 4). A group of section shapes widest at the top was named obpyriform, and two other groups of section shapes widest at the middle and bottom were designated as ellipse and pyriform, respectively. The classification tree enabled numerical determination of section shapes of the C2 lamina (Figure 3). If the ratio of WS to WI (WS/WI) was greater than 1.107 mm, the section shape was categorized as obpyriform. If WS/WI was within the range of 0.818 mm and 1.107 mm, the section shape was categorized as ellipse. On the basis of these criteria, 133 section shapes with WS/WI less than 0.818 mm remained unclassified, so the center position value was further selected to analyze section shape. In the group of 133 unclassified section shapes, if the center value was within the range of 56.72% and 58.28%, the section shape was classified as elliptical. The rest of the 124 section shapes were classified as pyriform.

The elliptical section shape was the most common shape, accounting for 52.07% (151 of 290 laminas) of the specimens, followed by the pyriform section shape (43.45%, 126 of 290 laminas). Thirteen of 290 laminar section shapes (4.48%) had an obpyriform shape. The center position values varied according to the type of cross-section shape (*p* < 0.001).

**Figure 5:**
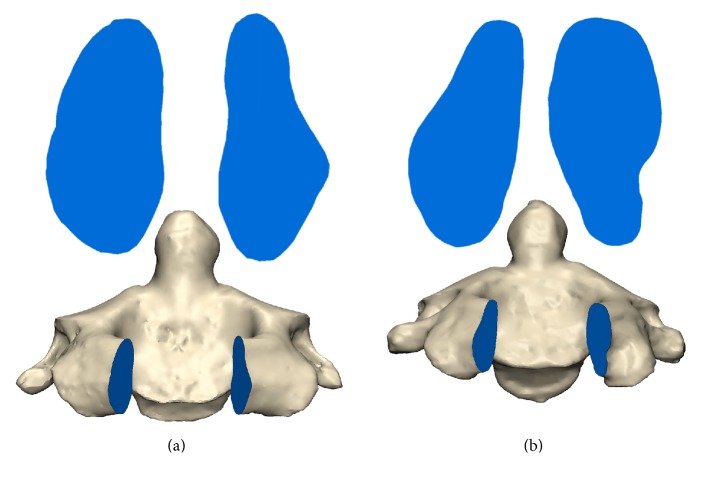
Asymmetric laminas (54 of 145 axes): (a) ellipse (L) and pyriform (R) (45 of 145 axes); (b) pyriform (L) and obpyriform (R) (9 of 145 axes).

Asymmetric laminas were found, in which the bilateral laminas were of different section shapes. Fifty-four of 145 axes showed this asymmetric pattern; of these 54 axes, 9 had an obpyriform section shape on either the left or right side (Figure 5).

## 4. Discussion

Since the introduction of translaminar screw fixation by Wright in 2004 [2], the morphometric characteristics of the C2 lamina have been widely studied to determine the suitability of the technique across different populations [8–11, 18, 26]. In particular, the measurement of thickness has been emphasized because it affects the maximum translaminar screw size. Small differences in thickness were noted among studies with values ranging from 5.17 ± 1.42 mm to 6.7 ± 1.5 mm [8–10, 12–18]. In the cadaveric study of Cassinelli et al. [8], the thickness of the lamina of 420 axis was 5.77 ± 1.31 mm. Similar studies conducted in the United States have identified laminar thicknesses of 6.3 ± 1.3 mm [9] and 5.5 ± 1.4 mm [18]. Kim et al. [10] found that laminar thickness in a sample of Korean patients was 5.66 ± 1.02 mm. In Chinese populations, the thickness of the lamina ranged from 5.87 ± 1.29 mm to 6.70 ± 1.50 mm [12–14]. The thickness values in Indian [16] and Malaysian [15] populations were measured relatively small as 5.17 ± 1.42 mm and 5.60 ± 1.20 mm, respectively. Saetia and Phankhongsab [17], on the other hand, reported the thickness as 6.64 ± 1.36 mm in a Thai population. These differences in thickness can be explained by different methodologies, including materials, specimens, measurement methods, or the definition of thickness.

Despite these differences, many researchers have concluded that most laminas can tolerate the placement of a 3.5 mm translaminar screw, assuming a tolerance margin of 1 mm [9–11, 14, 21] (Table 2). The current study also found that most specimens could safely accommodate a 3.5 mm translaminar screw. The lower and upper limits of the 95% confidence interval of laminar thickness were 6.26 mm and 6.54 mm, respectively, and 75.9% of laminas had thickness greater than 5.5 mm.

These linear measurements, however, only provide partial information on the lamina's 3D structure in the general population and fail to capture individual morphological differences. These thickness measurements cannot indicate the location of the thickest portion on the cross-section surface of the lamina. Ma et al. [13] and Sharma et al. [16] reported that the lamina is widest at the middle one-third. These results assume that the general shape of the laminar section is an ovoid. However, Senoglu et al. [11] showed that the lower one-third of the C2 lamina had the widest width, followed by the middle one-third and the upper one-third. In a morphometric study that assessed the cross-sectional area of the lamina [9], morphological diversity in cross-section shape was noted, although a detailed description was not provided. It can be assumed that, within the general population, thickness varies within individual lamina; for example, the lamina can have a much wider inferior than superior part and vice versa. However, there is limited data available regarding individual structural differences in the shape of the lamina.

The current guidelines for screw placement advise that the screw trajectory be directed toward the caudal portion of the lamina or parallel to the downslope of the lamina in order to avoid ventral breakout [2, 8, 14, 26]. Yue et al. [27] and Hu et al. [14] recommended the entry point of the screws be 5 mm posterior to the post-edge of the spinal canal. In terms of the screw angle, several studies measured the angles of the screw trajectory against the axis of the spinous process [8, 10, 12, 16, 26, 27]. The suggested screw angles, however, were varied between studies ranging from 42.45° to 59.19° and limited to the angles measured in the axial plane. Although a couple of researchers suggested angles measured in both axial and coronal planes [13, 14], these guidelines may not benefit those individuals having atypical C2 lamina structure and thus may increase the risk of cortical breach and potentially compromise biomechanical rigidity. Therefore, we analyzed the cross-sectional shape of the C2 lamina to confirm its structural diversity.

In the current study, 4.48% of the specimens (13 of 290 laminas) showed an obpyriform section shape, which previously has not been described in the literature. Of these 13 obpyriform-shaped laminas, five had thickness less than 5.5 mm, and the rest required translaminar screws to be placed in the cephalad half of the lamina in order to avoid cortical breach, which is inconsistent with the established advice. This finding demonstrates the importance of thorough preoperative planning using CT images, with additional consideration of the laminar section shape. In addition, although laminar asymmetry in relation to sectional surface was found in 37.24% of the studied specimens, none of the four morphometric measurements showed significant differences between sides. This discrepancy indicates that the asymmetric lamina is unlikely to be distinguished with a single measurement variable, but rather with the sectional shape criteria based on several measurement variables. Additional information on asymmetric section shapes could facilitate more efficient use of feasible laminas for translaminar screw placement, assisting surgeons in deciding which side should receive the screw superiorly.

## 5. Conclusion

This study provided morphometric data and analyzed structural variation in the C2 lamina in a Korean population, demonstrating the feasibility of translaminar screw fixation. The morphological analysis confirmed a distinctive “obpyriform” type of C2 laminar section shape in 4.48% of the studied specimens. Other common laminar section shapes were classified into pyriform and elliptical section shapes. Asymmetric laminas were noted in 37.24% of the studied C2 samples, which surgeons should consider when deciding screw placement order. The results of the current study are expected to enhance the current anatomical understanding of the C2 lamina in three dimensions. Additional consideration of the lamina's 3D structure during preoperative planning will enhance patient safety and the outcomes of translaminar screw fixation.

## Figures and Tables

**Table 1:** Morphometric parameters of the C2 lamina in the study population.

Parameters	Female (*N* = 130)	Male (*N* = 160)	Combined (*N* = 290)	*p*
Thickness (mm)	6.4 ± 1.3	7.1 ± 1.1	6.82 ± 1.2	<0.001
Diameter (mm)	6.0 ± 1.3	6.7 ± 1.1	6.39 ± 1.2	<0.001
Height (mm)	12.7 ± 1.4	14.1 ± 1.3	13.5 ± 1.5	<0.001
Center (%)	56.9 ± 7.2	57.7 ± 8.0	57.3 ± 7.7	0.356

**Table 2:** Comparison of morphometric parameters of the C2 lamina across studies.

	Population	Specimens (number of axes)	Thickness (mm)	Thickness >5.5 mm (%)	95% CI (lower-upper)
*Current study*	*Korean*	*145* *(CT scans)*	*6.39 ± 1.21*	*75.9*	*6.26–6.54*

Cassinelli et al. (2006)	American	420	5.77 ± 1.31	70.5 (>5 mm)	Null

Wang (2006)	American	38 (Cadavers)	6.30 ± 1.30	79	Null

Kim et al. (2008)	Korean	102 (CT scans)	5.66 ± 1.02	61.3	Null

Ma et al. (2010)	Chinese	120 (Cadavers)	5.87 ± 1.29	83.3 (≥4 mm)	Null

Bhatnagar et al. (2010)	American	50	5.50 ± 1.40	94	Null

Hu et al. (2010)	Chinese (Han)	28	6.70 ± 1.50	“Bulk”	Null

Xin-Yu et al. (2011)	Chinese	96 (Cadavers)	6.20 ± 5.20	85 (>5 mm)	Null
		112 (CT scans)	6.60 ± 1.50		

Yusof and Shamsi (2012)	Malaysian	98 (CT scans)	5.60 ± 1.20	75.5 (≥5 mm)	Null

Sharma et al. (2015)	Indian	38 (Cadavers)	5.17 ± 1.42	36.9	Null
		(CT scans)	5.57 ± 1.28	NA	

Saetia and Phankhongsab (2015)	Thai	200	6.64 ± 1.36	79	Null
